# Non-pharmaceutical interventions and risk of COVID-19 infection: survey of U.K. public from November 2020 – May 2021

**DOI:** 10.1186/s12889-023-15209-6

**Published:** 2023-02-24

**Authors:** Nick A. Francis, Taeko Becque, Merlin Willcox, Alastair D. Hay, Mark Lown, Richard Clarke, Beth Stuart, Lucy Yardley, Michael Moore, Joëlle Houriet, Paul Little

**Affiliations:** 1 Primary Care Research Centre, School of Primary Care, Population Sciences and Medical Education, Faculty of Medicine, NIHR School for Primary Care Research, University of Southampton, Aldermoor Health Centre, Southampton, SO16 5ST UK; 2 Centre for Academic Primary Care, Bristol Medical School: Population Health Sciences, NIHR School for Primary Care Research, University of Bristol, 39 Whatley Road, Bristol, BS8 2PS UK; 3 School of Natural and Social Sciences, University of Gloucestershire, Francis Close Hall, Swindon Road, Cheltenham, GL50 4AZ UK; 4 Pragmatic Clinical Trials Unit, Queen Mary University of London, Yvonne Carter Building, 58 Turner Street, London, E1 2AB UK; 5 School of Psychology, University of Southampton, Highfield Campus, Southampton, SO17 1BJ UK; 6 School of Psychological Science, University of Bristol, 12A Priory Road, Bristol, BS8 1TR UK; 7 Antenna Foundation, Avenue de La Grenade 24, 1207 Geneva, Switzerland

**Keywords:** COVID-19, Non-pharmaceutical interventions, Face covering, Social distancing, Handwashing, Risk

## Abstract

**Introduction:**

Non-pharmaceutical interventions (NPIs), such as handwashing, social distancing and face mask wearing, have been widely promoted to reduce the spread of COVID-19. This study aimed to explore the relationship between self-reported use of NPIs and COVID-19 infection.

**Methods:**

We conducted an online questionnaire study recruiting members of the UK public from November 2020 to May 2021. The association between self-reported COVID-19 illness and reported use of NPIs was explored using logistic regression and controlling for participant characteristics, month of questionnaire completion, and vaccine status. Participants who had been exposed to COVID-19 in their household in the previous 2 weeks were excluded.

**Results:**

Twenty-seven thousand seven hundred fifty-eight participants were included and 2,814 (10.1%) reported having a COVID-19 infection. The odds of COVID-19 infection were reduced with use of a face covering in unadjusted (OR 0.17 (95% CI: 0.15 to 0.20) and adjusted (aOR 0.19, 95% CI 0.16 to 0.23) analyses. Social distancing (OR 0.27, 95% CI: 0.22 to 0.31; aOR 0.35, 95% CI 0.28 to 0.43) and handwashing when arriving home (OR 0.57, 95% CI 0.46 to 0.73; aOR 0.63, 95% CI: 0.48 to 0.83) also reduced the odds of COVID-19. Being in crowded places of 10–100 people (OR 1.89, 95% CI: 1.70 to 2.11; aOR 1.62, 95% CI: 1.42 to 1.85) and > 100 people (OR 2.33, 95% CI: 2.11 to 2.58; aOR 1.73, 95% CI: 1.53 to 1.97) were both associated with increased odds of COVID-19 infection. Handwashing before eating, avoiding touching the face, and cleaning things with virus on were all associated with increased odds of COVID-19 infections.

**Conclusions:**

This large observational study found evidence for strong protective effects for individuals from use of face coverings, social distancing (including avoiding crowded places) and handwashing on arriving home on developing COVID-19 infection. We also found evidence for an increased risk associated with other behaviours, possibly from recall bias.

**Supplementary Information:**

The online version contains supplementary material available at 10.1186/s12889-023-15209-6.

## Background

More than two years into the pandemic, COVID-19 continues to cause widespread disruption and costs for societies around the globe [1]. Although vaccination against SARS-CoV-2 has started to transform the impact of the COVID-19 pandemic, many countries continue to have sub-optimal vaccination coverage and post-vaccination breakthrough illnesses are increasing. Furthermore, new variants such as Omicron pose an ongoing threat. Therefore, there is still a need to understand the role of non-pharmaceutical interventions (NPIs) such as face covering, social distancing, handwashing and cleaning surfaces.

SARS-CoV-2 can be transmitted by symptomatic and asymptomatic individuals [2], with respiratory transmission now widely thought to be the main route of transmission [3]. However, there is also clear evidence that proximity is a risk factor [4], suggesting that respiratory droplets may be more important than aerosols. The evidence for fomite transmission is much less strong [3]. However, some studies have found evidence for an increased risk in those with poor hand hygiene and reduced risk associated with regular use of disinfectants [5]. There is evidence that SARS-CoV-2 can infect domestic pets [6], but no confirmed cases of transmission from pets to humans.

In the U.K., a full lockdown was announced on 23^rd^ March 2020 and became legally binding 3 days later. There was a phased easing of lockdown from 1^st^ June 2020 but localised lockdowns in areas with high levels of infection continued for several months. PCR testing for SARS-COV-2 was initially restricted to hospital inpatients. However, in May 2020 a national “test and trace” system was set up, offering free PCR testing for anyone with symptoms in the community. Walk-in and drive-through test sites were gradually set up over the next few months and by mid-September 2020 more than 11% of people living in England had been tested. Face coverings became compulsory in shops and other indoor public places from 24^th^ July 2020. A second national lockdown came into force in England from 5^th^ November to 2^nd^ December 2020, and a third from 6^th^ January to 29^th^ March 2021. The national vaccination programme started in January 2021, prioritising the most vulnerable people first. “Non-essential shops” and outdoor restaurants were allowed to reopen from 12^th^ April 2021. All lockdown measures were lifted on 19^th^ July 2021 [7].

At the start of the pandemic, recommendations for NPI were based on evidence from other respiratory viruses such as influenza. The impact of different NPIs was unclear and there continues to be debate about their effectiveness. All legal requirements to wear face coverings have now been lifted in the U.K. However, the World Health Organisation, European Centre for Disease Control and U.S. Centres for Disease Control and Prevention all still promote the use of face masks, social distancing and hand washing to prevent the spread of COVID-19. Systematic review of the evidence for social distancing and mask wearing suggest that both are likely to be important but the evidence is limited by small studies, the difficulty in controlling for confounding, and/or were based in secondary care settings or used atypical community sample (e.g. high risk gatherings, travellers) [4, 8, 9]. To better inform public health advice, we set out to explore the relationship between self-reported use of NPIs and COVID-19 infection using data from a large international survey study. The aim was to explore the protective effect of NPIs, such as handwashing and wearing a face mask, on the risk of developing COVID-19 illness by describing the association between self-reported individual use of NPIs and COVID-19 infection.

## Methods

For this study we used data from the U.K. RTO-COVID-19 survey. This was a large international online survey designed to explore associations between preventive measures and incidence of COVID-19, as well as associations between treatments and outcomes (Retrospective Treatment and Outcomes study on COVID-19: RTO-COVID-19).

Data from other countries were not used for this analysis, as the time frame for national preventive measures and questions about NPIs were different.

### Participant recruitment

The study opened to recruitment in the U.K. in July 2020 and concluded in July 2021. In November 2020, we changed the questions about NPIs to ask about behaviours ‘during the last two weeks’ instead of ‘during the lockdown’. Therefore, for this study we have only used data from participants who completed the survey following this change in November 2020. Participants joined the study by completing an online questionnaire developed using LimeSurvey and hosted by the University of Geneva. The study was open to anyone aged 16 or older who had capacity to consent for themselves and was willing to participate. Several approaches were used to invite people to participate in the study. We initially used social media and contacted organisations such as parish councils, religious organisations, and sports clubs, and asked them to send information about the study to their members. We also sent invitations to staff at academic institutions and personal contacts of the study team, and asked them to forward the information to their contacts (snowballing). Finally, in March 2021 we obtained permission to recruit general practices who sent invitations by SMS text message to adult patients registered with them. Over 848,000 text message invitations were sent by 116 practices in all parts of England.

### Questionnaires

The questionnaire included socio-demographic data, history of longstanding physical and mental health conditions, use of regular medications, history of respiratory tract infections since the start of the pandemic, and further information about the worst of these illness. Participants were also asked about use of NPIs during the two weeks before their illness (all participants who reported having a respiratory tract infection since the start of the lockdown) and two weeks prior to completing their questionnaire (all other participants from November 2020 onwards).

### Study populations and case definitions

Our populations included all participants who completed the survey from November 2020 until the end of June 2021 (when data collection finished). However, participants were invited to report respiratory tract infections that occurred from the 1^st^ January 2020. For all analyses, participants were excluded if they had a household member who had an acute respiratory infection in the two weeks before they became ill (for participants who had COVID-19) or at any point from the start of the pandemic until they completed the questionnaire (for those who did not have COVID-19). The effect of NPIs in those who had a household contact are likely to be different and will be explored in a separate analysis.

Case definitions used in the primary analysis and sensitivity analyses are detailed in Table 1.

**Table 1:** Analyses, study populations and case definitions

Analysis	Cases^a^	Comparison group	Time window for NPIs
Primary	Probable COVID-19	Study population excluding cases and those meeting our broader ‘Suspected COVID-19’ definition	2 weeks before questionnaire completion (cases and comparison)
Sensitivity 1	Probable COVID-19, excluding those who reported receiving one or more COVID-19 vaccine doses prior to completing the questionnaire	Study population excluding cases, ‘Suspected COVID-19’, and participants who reported receiving one or more COVID-19 vaccine doses prior to completing the questionnaire	2 weeks before questionnaire completion (cases and comparison)
Sensitivity 2	Suspected COVID-19	Those not meeting case definition	2 weeks before questionnaire completion (cases and comparison)
Sensitivity 3	Symptomatic COVID-19	Those not meeting case definition	2 weeks before illness (cases) and 2 weeks before questionnaire completion (comparison group)
Sensitivity 4	Any acute RTI lasting 3 days or more	Those not meeting case definition	2 weeks before illness (cases) and 2 weeks before questionnaire completion (comparison group)

^a^Probable COVID-19: Positive COVID-19 ‘nose or throat swab’ test AND/OR self-reported respiratory illness (of 3 or more days) occurring during the pandemic period that was associated with fever and loss of smell and/or taste [10]

Suspected COVID-19: Positive COVID-19 ‘nose or throat swab’ test AND/OR self-reported respiratory illness (of 3 or more days) occurring during the pandemic period that was associated with loss or smell and/or taste OR fever and cough OR three or more of: fever, cough, fatigue, headache, myalgia, sore throat, runny or blocked nose, shortness of breath, nausea or vomiting, diarrhoea [11]

Symptomatic COVID-19: Those meeting the criteria for Susptected COVID-19, but excluding those with a positive nose or throat swab who did not report having a respiratory illness lasting 3 days or more

For our primary analysis we defined “probable COVID-19” as those who reported a positive COVID-19 ‘nose or throat swab’ test (most cases occurred before use of lateral flow tests was common) or had a self-reported respiratory illness occurring during the pandemic period that was associated with fever and loss of smell and/or taste. We wanted to have a primary case definition that was specific, but as we included participants who reported illnesses at a time when testing for SARS-CoV-2 in the community was uncommon we included a symptom-based definition that was relatively specific during this high-prevalence period [10].

We included four sensitivity analyses to explore the robustness of our findings. The first excluded those who reported having received one or more COVID-19 vaccination doses prior to completing the questionnaire. COVID-19 vaccination is the most effective preventive measure and therefore receipt of a vaccination could affect both risk of infection and perceptions of risk and therefore behaviours. The second used a broader ‘Suspected COVID-19’ definition based on the WHO case definition [11] The third sensitivity analysis included only participants who reported a symptomatic episode meeting our definition of probable or suspected COVID-19 (excluding those who had a positive test for COVID-19 but did not report having a respiratory illness lasting 3 days or more). In this analysis we had an illness date for all cases and were therefore able to compare NPIs reported in the two weeks prior to illness (cases) with NPIs in the two weeks prior to questionnaire completion (comparison group). The fourth sensitivity analysis used all participants who reported an acute respiratory tract infection (ARI) for 3 days or more as the case definition.

### Exposures

The exposures of interest in this study were: 1) washing hands with soap or alcohol gel when coming home, 2) washing hands with soap or alcohol gel before eating, 3) maintaining social distancing (2 m or more) from those outside their house, 4) avoiding touching the face, 5) cleaning things that might have virus on them (e.g. doors, taps), 6) wearing a face mask or face covering, 7) avoiding touching other people’s pets, 8) using other approaches (such as diets, vitamins, nasal sprays, medicines). Frequency of these exposures was assessed using a five-point Likert scale: ‘Never (or almost never)’, ‘Sometimes’, ‘Quite often’, ‘Very often’, ‘Always (or almost always)’, ‘Don’t know’ or ‘Not applicable’. We also asked participants to rate how often they had been in crowded places for 15 min or more in the past 2 weeks (defined as 10–100 people and over 100 people), using the categories ‘Never, ‘1–2 times’, ‘3–4 times’, ‘5–6 times’, ‘7–9 times’,’10 or more times’.

Both groups of questions were analysed in two ways: a) comparing ‘Never’ with any use of the NPI (i.e. all other categories), and b) including them as continuous variables in order to assess the effect of moving from one level to the next. It is worth noting that the first set of behaviours are NPIs that are expected to be protective and associated with reduced risk of COVID-19, while the last two (being in crowded places) are expected to be risk factors and associated with increased risk of COVID-19.

### Analysis plan

We calculated Spearman correlation coefficients between reported use of each NPI. We then developed logistic regression models with our case definition as the dependent variable. We initially assessed the univariable association between each exposure variable and the outcome. We then developed multivariable regression models in three stages: 1) controlling for demographics (age, gender, ethnicity, socioeconomic status), month of questionnaire completion, and vaccination status (no doses before questionnaire completion, one dose before questionnaire completion, two doses with second dose being less than 28 days before questionnaire completion, and two doses with second dose being 28 days or more before questionnaire completion); 2) adding in other potential confounders (money problems; working outside home; number of people in the household (lives alone, lives with one other person, 3–6 people in the house, more than 6 people in the house); having pets; pregnancy (assuming missing not pregnant); number of comorbid conditions; history of a mental health problem; self-reported regular use of steroids or immunosuppressant medication; statins; medications for diabetes; self-reported weight (normal, underweight, overweight); smoking status; anxiety (from PHQ-4); depression (from PHQ-4); U.K. region; and month of questionnaire completion interacted with region); 3) adding in all other NPIs (and exposure to crowded places). Model 2 (adjusted for all potential confounders but not other NPIs) was considered to be our primary adjusted analysis.

For the sensitivity analyses we only included behaviours as continuous variables and only present unadjusted and model 1 results.

## Results

**Fig. 1:**
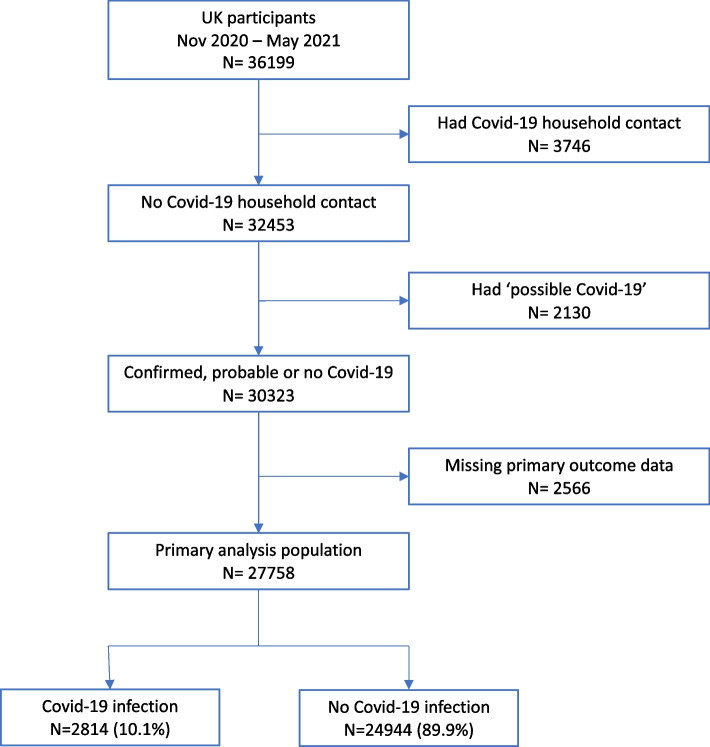
Participant flow diagram

Thirty-six thousand one hundred ninety-nine participants completed the survey between November 2020 and June 2021. Participant inclusion is summarised in Fig. 1. We excluded 3,746 (10.3%) who had a household member with an ARI in the two weeks before they became ill (for participants who had COVID-19) or at any point from the start of the pandemic until they completed the questionnaire (for those who did not have COVID-19), 2,130 (5.9%) people who did not meet our definition of COVID-19, but did meet our definition of ‘suspected COVID-19’ and 2,565 (7.1%) who had missing data on the primary outcome. This left a primary analysis sample of 27,758, of whom 2,814 (10.1%) had “probable COVID-19”. Of those with probable COVID-19, 1074 (38.2%) had a positive COVID-19 test and reported having symptoms of an ARI lasting 3 days or more, 1175 (41.8%) had a positive test but did not report having an ARI for 3 days or more, and 565 (20.1%) did not report a positive COVID-19 test but did report symptoms that met our definition of probable COVID-19. Characteristics of the primary analysis population are given in Tables 2 and 3. The characteristics of the excluded populations and the population with COVID-19 but not reporting an ARI lasting 3 days or more were similar to the primary analysis population (Supplementary Table 1).

**Table 2:** Demographic characteristics of primary analysis population

	Probable COVID-19 infection		No COVID-19		Overall	
	n	%	n	%	n	%
Gender						
Female	1933	69.3	15,131	61.2	17,064	62.0
Male	858	30.7	9609	38.8	10,467	38.0
Total (n, % response)	2791 (99.2%)	100.0	24,740 (99.2%)	100.0	27,531 (99.2%)	100.0
Age group						
< 25	254	9.1	1298	5.2	1552	5.6
25–49	1250	44.8	7504	30.3	8754	31.8
50–64	960	34.4	8084	32.7	9044	32.8
65–79	312	11.2	7243	29.3	7555	27.4
80 +	15	0.5	624	2.5	639	2.3
Total (n, % response)	2791 (99.2%)	100.0	24,753 (99.2%)	100.0	27,544 (99.2%)	100.0
Ethnicity						
White	2000	88.1	21,169	92.8	23,169	92.3
Black	62	2.7	327	1.4	389	1.6
South Asian	136	6.0	616	2.7	752	3.0
Chinese	6	0.3	113	0.5	119	0.5
Arab	9	0.4	61	0.3	70	0.3
Mixed	31	1.4	325	1.4	356	1.4
Don't know	7	0.3	15	0.1	22	0.1
No answer	8	0.4	59	0.3	67	0.3
Other	12	0.5	139	0.6	151	0.6
Total (n, % response)	2271 (80.7%)	100.0	22,824 (91.5%)	100.0	25,095 (90.4%)	100.0
Region						
Greater London	179	6.4	1168	4.7	1347	4.9
East Midlands	275	9.8	2259	9.1	2534	9.1
East of England	23	0.8	200	0.8	223	0.8
North East	37	1.3	416	1.7	453	1.6
North West	171	6.1	1164	4.7	1335	4.8
South East	474	16.8	5406	21.7	5880	21.2
South West	381	13.5	5648	22.6	6029	21.7
Yorkshire and the Humber	287	10.2	3048	12.2	3335	12.0
West Midlands	272	9.7	1792	7.2	2064	7.4
Scotland	27	1.0	422	1.7	449	1.6
Wales	40	1.4	320	1.3	360	1.3
Other or missing	648	23.0	3101	12.4	3749	13.5
Total	24,944	100	2814	100	27,758	100
Money problems						
No problems	1343	59.3	16,811	73.9	18,154	72.6
Some problems	755	33.3	4921	21.7	5676	22.7
Big problems	109	4.8	699	3.1	808	3.2
Huge problems	58	2.6	304	1.3	362	1.5
Total (n, % response)	2265 (80.5%)	100.0	22,735 (91.1%)	100.0	25,000 (90.1%)	100.0
Work outside home						
No	1466	64.4	19,030	83.2	20,496	81.5
Yes	811	35.6	3832	16.8	4643	18.5
Total (n, % response)	2277 (80.9%)	100.0	22,862 (91.7%)	100.0	25,139 (90.6%)	100.0
Number in household						
Live alone	661	33.1	7852	43.7	8513	42.7
Live with one other	387	19.4	4225	23.5	4612	23.1
3–6 people	818	40.9	5328	29.7	6146	30.8
7 or more	132	6.6	558	3.1	690	3.5
Total (n, % response)	1998 (71.0%)	100.0	17,963 (72.0%)	100.0	19,961 (71.9%)	100.0
Pets in household						
Yes	1079	47.9	9517	42.3	10,596	42.8
No	1174	52.1	12,986	57.7	14,160	57.2
Total (n, % response)	2253 (80.0%)	100.0	22,503 (90.2%)	100.0	24,756 (89.2%)	100.0
Month questionnaire completed						
November 2020	56	2.0	978	3.9	1034	3.7
December 2020	46	1.6	389	1.6	435	1.6
January 2021	231	8.2	1550	6.2	1781	6.4
February 2021	61	2.2	399	1.6	460	1.7
March 2021	518	18.4	4746	19.0	5264	19.0
April 2021	1775	63.1	15,806	63.4	17,581	63.3
May 2021	127	4.5	1076	4.3	1203	4.3
Total	2814	100.0	24,944	100.0	27,758	100.0
	N (% response)	Median, IQR	N (% response)	Median, IQR	N (% response)	Median, IQR
Self-rated socio-economic status^a^	2227 (79.1%)	5 (4, 6)	22,323 (89.5%)	5 (4, 7)	24,583 (88.6%)	5 (4, 7)

^a^Self-reported socio-economic status on a scale 1 (lowest) to 10 (highest)

**Table 3:** Health characteristics of primary analysis population

	Probable COVID-19 infection		No COVID-19		Total	
	n	%	n	%	n	%
Pregnant^a^						
No	2743	97.6	24,480	98.3	27,223	98.2
Yes	67	2.4	428	1.7	495	1.8
Total (n, % response)	2810 (99.9%)	100.0	24,908 (99.9%)	100.0	27,718 (99.9%)	100.0
Mental health problem						
No	2391	85.0	21,678	86.9	24,069	86.7
Yes	423	15.0	3265	13.1	3688	13.3
Total (n, % response)	2814 (100%)	100.0	24,943 (100.0)	100.0	27,757 (100.0)	100.0
Immunosuppressant medication						
No	2748	97.7	24,109	96.7	26,857	96.8
Yes	65	2.3	833	3.3	898	3.2
Total (n, % response)	2813 (100%)	100.0	24,942 (100%)	100.0	27,755 (100%)	100.0
Statins						
No	2523	89.7	20,308	81.4	22,831	82.3
Yes	290	10.3	4634	18.6	4924	17.7
Total (n, % response)	2813 (100%)	100.0	24,942 (100%)	100.0	27,755 (100%)	100.0
Diabetes medication						
No	2679	95.2	23,450	94.0	26,129	94.1
Yes	134	4.8	1492	6.0	1626	5.9
Total (n, % response)	2813 (100%)	100.0	24,942 (100%)	100.0	27,755	100.0
Self-reported weight						
Underweight	60	2.7	604	2.7	664	2.7
Normal weight	1167	52.3	11,779	52.9	12,946	52.9
Overweight	1006	45.1	9867	44.4	10,873	44.4
Total (n, % response)	21,439 (87.4%)	100.0	569 (71.8%)	100.0	22,008 (86.9%)	100.0
Smoking status						
No smoking	1870	84.7	18,945	86.2	20,815	86.1
Tobacco	223	10.1	2201	10.0	2424	10.0
E-cigarette	115	5.2	834	3.8	949	3.9
Total (n, % response)	2208 (78.5%)	100.0	21,980 (88.1%)	100.0	24,188 (87.1%)	100.0
Anxiety (PHQ-4)						
No	1723	77.2	18,892	85.2	20,615	84.4
Yes	510	22.8	3289	14.8	3799	15.6
Total (n, % response)	2233 (79.4%)	100.0	22,181 (88.9%)	100.0	24,414 (88.0%)	100.0
Depression (PHQ-4)						
No	1722	77.9	18,883	86.0	20,605	85.3
Yes	488	22.1	3075	14.0	3563	14.7
Total (n, % response)	2210 (78.5%)	100.0	21,958 (88.0%)	100.0	24,168 (87.1%)	100.0
Vaccination status						
None	667	30.4	4617	21.3	5284	22.1
Only one dose	3	0.1	27	0.1	30	0.1
Second vaccine dose 4 weeks or less before questionnaire completion	336	15.3	5008	23.1	5344	22.4
Second dose more than 4 weeks before window	1190	54.2	12,064	55.6	13,254	55.4
Total (n, % response)	2196 (78.0%)	100.0	21,716 (87.1%)	100.0	23,912 (86.1%)	100.0
	N, % response	Median, IQR	N, % response	Median, IQR	N, % response	Median, IQR
Number of health conditions	2814 (100%)	0 (0,1)	24,944 (100%)	0 (0,1)	27,758 (100%)	0 (0,1)

^a^In analysis, missing pregnancy is assumed to be not pregnant

**Fig. 2:**
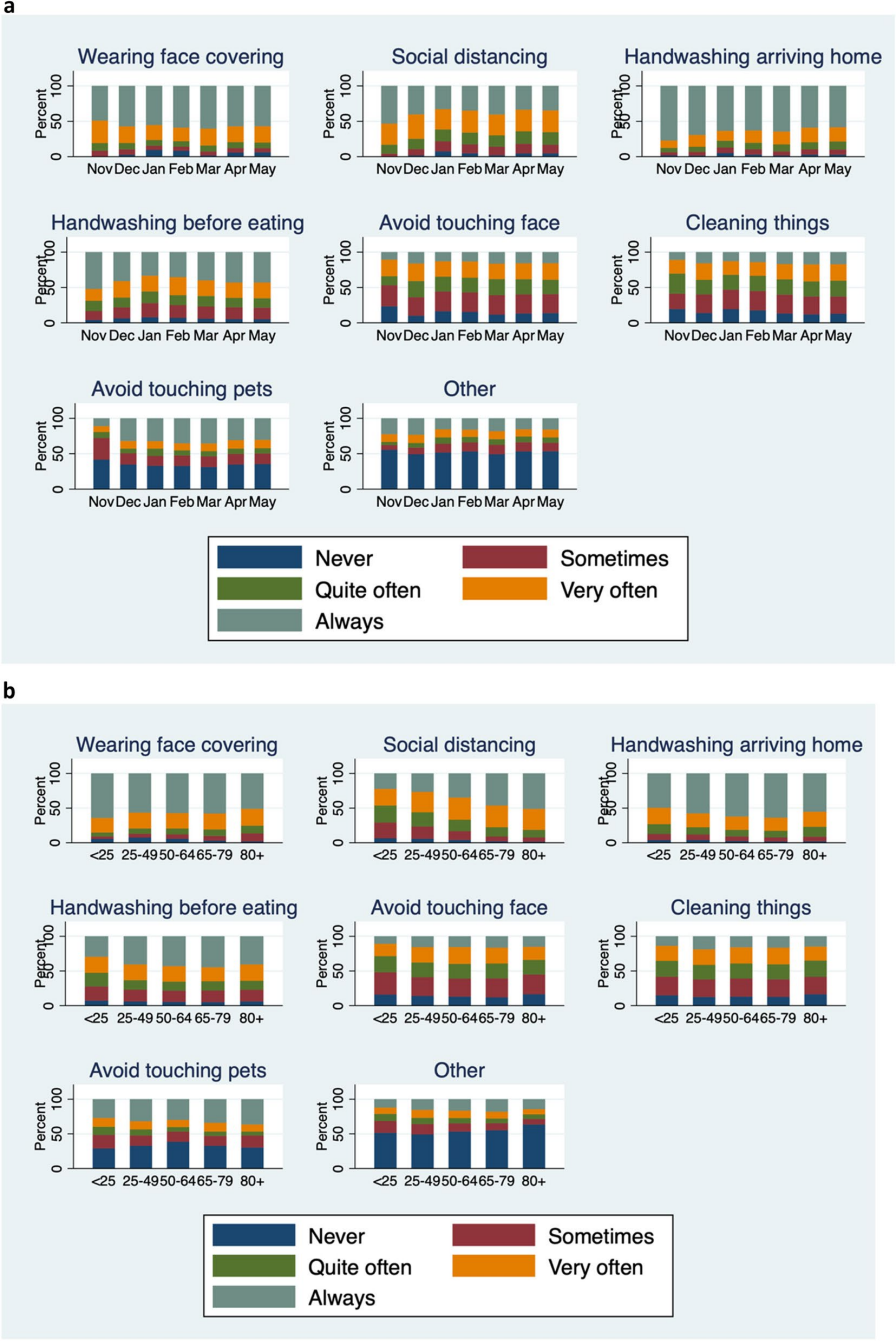
**a** Preventive behaviours by month of questionnaire completion. **b** Preventive behaviours by age group

**Fig. 3:**
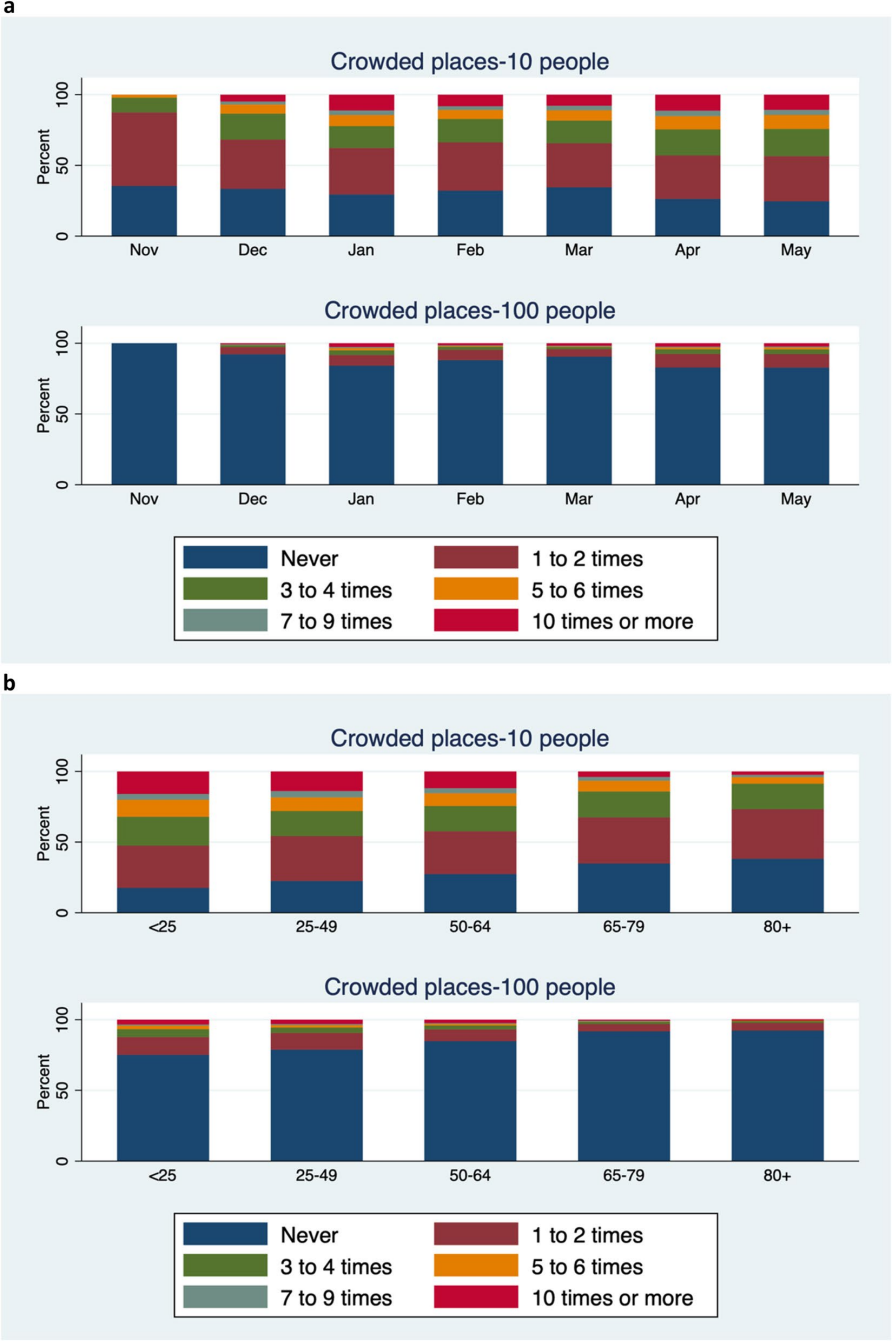
**a** Frequency of being in crowded places by month of questionnaire completion. **b** Frequency of being in crowded places by age group

Reported use of NPIs by month of questionnaire completion and age group are shown in Fig. 2a and b respectively, and by COVID-19 status in Table 4. Reported use of social distancing and handwashing reduced between November 2020 and May 2021, but use of the other behaviours stayed fairly constant. Most NPIs were used slightly more frequently with increasing age, and this was particularly true for social distancing. Reported frequency of being in crowded places, by month of questionnaire completion and age group are shown in Fig. 3a and b respectively, and by COVID-19 status in Table 5. There was a trend of increasing attendance at crowded places over the study period and a strong trend of reducing attendance at crowded places with increasing age. Most NPIs were moderately correlated, with the correlation between NPIs and going into crowded places (risk activity) being negatively correlated, as expected (Table 6).

**Table 4:** Use of non-pharmaceutical interventions (NPIs)

NPI	COVID-19 infection	Never	Sometimes	Quite often	Very often	Always
Wearing a face covering	Yes (*n* = 2326)	284 (12.2)	157 (6.8)	198 (8.5)	494 (21.2)	1193 (51.3)
	No (*n* = 23,404)	540 (2.3)	1369 (5.9)	1995 (8.5)	5477 (23.4)	14,023 (59.9)
Social distancing	Yes (*n* = 2331)	197 (8.5)	418 (17.9)	384 (16.5)	659 (28.3)	673 (28.9)
	No (*n* = 23,357)	560 (2.4)	2830 (12.1)	4117 (17.6)	7400 (31.7)	8450 (36.2)
Handwashing when arriving home	Yes (*n* = 2371)	86 (3.6)	204 (8.6)	310 (13.1)	541 (22.8)	1230 (51.9)
	No (*n* = 23,473)	497(2.1)	1449 (6.2)	2267 (9.7)	4574 (19.5)	14,686 (62.6)
Handwashing before eating	Yes (*n* = 2361)	83 (3.5)	307 (13.0)	324 (13.7)	542 (23.0)	1105 (46.8)
	No (*n* = 23,371)	1323 (5.7)	4003 (17.1)	3171 (13.6)	5169 (22.1)	9705 (41.5)
Avoid touching face	Yes (*n* = 2304)	248 (10.8)	547 (23.7)	504 (21.9)	579 (25.1)	426 (18.5)
	No (*n* = 23,041)	2953 (12.8)	6296 (27.3)	4976 (21.6)	5267 (22.9)	3549 (15.4)
Cleaning things	Yes (*n* = 2337)	209 (8.9)	452 (19.3)	471 (20.2)	582 (24.9)	623 (26.7)
	No (*n* = 23,346)	3024 (13.0)	6093 (26.1)	5101 (21.9)	5436 (23.3)	3692 (15.8)
Avoid touching others’ pets	Yes (*n* = 1811)	395 (21.8)	222 (12.3)	158 (8.7)	209 (11.5)	827 (45.7)
	No (*n* = 19,786)	6995 (35.4)	3057 (15.5)	1473 (7.4)	2316 (11.7)	5945 (30.1)
Other	Yes (*n* = 2126)	852 (40.1)	315 (14.8)	242 (11.4)	291 (13.7)	426 (20.0)
	No (*n* = 22,146)	11,927 (53.9)	2707 (12.2)	1704 (7.7)	2296 (10.4)	3512 (15.9)

**Table 5:** Frequency of attendance at crowded places

	**COVID-19 infection**	**Never**	**1–2 times**	**3–4 times**	**5–6 times**	**7–9 times**	**10 + times**
Crowded places of 10 -100 people	Yes (*n* = 2385)	434 (18.2)	621 (26.0)	389 (16.3)	274 (11.5)	134 (5.62)	533 (22.35)
	No (*n* = 23,643)	7004 (29.6)	7739 (32.7)	4361 (18.5)	1998 (8.5)	735 (3.1)	1806 (7.6)
Crowded places of more than 100 people	Yes (*n* = 2287)	1698 (74.3)	287 (12.6)	97 (4.2)	58 (2.5)	35 (1.5)	112 (4.9)
	No (*n* = 23,574)	20,523 (87.1)	1836 (7.8)	580 (2.5)	214 (0.9)	91 (0.4)	330 (1.4)

**Table 6:** Correlation between NPIs

	Wearing a mask	Social distancing	Handwashing when arriving home	Handwashing before eating	Avoid touching face	Cleaning things	Avoid touching others’ pets	Other	Been in crowded places 10 people
Wearing a mask	1								
Social distancing	0.26	1							
Handwashing when arriving home	0.29	0.23	1						
Handwashing before eating	0.22	0.19	0.50	1					
Avoid touching face	0.29	0.29	0.38	0.42	1				
Cleaning things	0.31	0.26	0.38	0.47	0.50	1			
Avoid touching others’ pets	0.19	0.19	0.23	0.25	0.32	0.34	1		
Other	0.13	0.12	0.17	0.20	0.24	0.29	0.26	1	
Been in crowded places 10 people	-0.06	-0.29	-0.08	-0.06	-0.09	-0.09	-0.10	-0.06	1
Been in crowded places 100 people	-0.07	-0.15	-0.09	-0.02	-0.04	-0.01	-0.03	-0.00	0.30

Data in this table are Spearman correlation coefficients for reported NPIs

### Primary analysis

Estimates of the associations between NPIs and COVID-19 infection, as well as the associations between being in crowded places and COVID-19 infection, in unadjusted and adjusted models are shown in Tables 7 and 8. Use of a face covering, social distancing and handwashing when arriving home were all associated with a reduced odds of COVID-19 infection in unadjusted and all adjusted models. Use of a face covering was associated with the largest reduction in odds of COVID-19 (OR 0.19, 95% CI: 0.16 to 0.23 for any (versus no) use of face coverings, and OR 0.73, 95% CI: 0.70 to 0.76 for each increase in level of use) in adjusted analyses, followed by social distancing (OR 0.35, 95% CI: 0.28 to 0.43 for any use of social distancing and OR 0.88, 95% CI: 0.84 to 0.92 for each increase in level of use, adjusted analyses) and then handwashing when arriving home (0.63, 95% CI: 0.48 to 0.83 for any use and OR 0.84, 95% CI, 0.80 to 0.88) for each increase in level of use, adjusted analyses).

**Table 7:** Association between any use of non-pharmaceutical interventions (NPIs), and any exposure to crowded places, and COVID-19 Illness

	**Unadjusted**		**Model 1**		**Model 2**		**Model 3**	
	**N**	**OR (95% CI)**	**N**	**OR (95% CI)**		**OR (95% CI)**	**N**	**OR (95% CI)**
Wearing a mask	25,730	**0.17 (0.15 to 0.20)**	22,297	**0.19 (0.16 to 0.22)**	16,843	**0.19 (0.16 to 0.23)**	12,566	**0.16 (0.12 to 0.21)**
Social distancing	25,688	**0.27 (0.22 to 0.31)**	22,257	**0.31 (0.26 to 0.38)**	16,803	**0.35 (0.28 to 0.43)**	12,566	**0.58 (0.41 to 0.81)**
Handwashing when arriving home	25,844	**0.57 (0.46 to 0.73)**	22,381	**0.65 (0.50 to 0.84)**	16,900	**0.63 (0.48 to 0.83)**	12,566	0.71 (0.46 to 1.09)
Handwashing before eating	25,732	**1.65 (1.31 to 20.6)**	22,292	**1.65 (1.29 to 2.10)**	16,849	**1.49 (1.14 to 1.94)**	12,566	1.15 (0.79 to 1.66)
Avoid touching face	25,345	**1.22 (1.06 to 1.40)**	21,969	**1.18 (1.02 to 1.37)**	16,636	1.17 (0.99 to 1.38)	12,566	1.17 (0.91 to 1.52)
Cleaning things	25,683	**1.52 (1.31 to 1.76)**	22,270	**1.39 (1.19 to 1.63)**	16,819	**1.38 (1.15 to 1.64)**	12,566	**1.39 (1.06 to 1.81)**
Avoid touching others’ pets	21,597	**1.96 (1.75 to 2.20)**	18,726	**2.00 (1.77 to 2.27)**	14,066	**2.12 (1.84 to 2.44)**	12,566	**2.41 (2.02 to 2.88)**
Other	24,272	**1.75 (1.59 to 1.91)**	21,031	**1.59 (1.44 to 1.76)**	15,879	**1.62 (1.45 to 1.81)**	12,566	**1.53 (1.32 to 1.76)**
Been in crowded places 10 people	26,028	**1.89 (1.70 to 2.11)**	22,556	**1.70 (1.51 to 1.92)**	17,027	**1.62 (1.42 to 1.85)**	12,566	**1.41 (1.19 to 1.68)**
Been in crowded places 100 people	25,861	**2.33 (2.11 to 2.58)**	22,401	**1.87 (1.67 to 2.09)**	16,897	**1.73 (1.53 to 1.97)**	12,566	**1.40 (1.18 to 1.66)**

Model 1: controlling for demographics (age, gender, ethnicity, socioeconomic status), month of questionnaire completion, and vaccination status

Model 2: as per model 1 plus controlling for money problems; working outside home; number of people in the household (lives alone, lives with one other person, 3–6 people in the house, more than 6 people in the house); having pets; pregnancy (assuming missing not pregnant); number of comorbid conditions; history of a mental health problem; self-reported regular use of steroids or immunosuppressant medication; statins; medications for diabetes; self-reported weight (normal, underweight, overweight); smoking status; anxiety (from PHQ-4); depression (from PHQ-4); U.K. region; and month of questionnaire completion interacted with region

Model 3: as per model 2 plus controlling for other NPIs and being in crowded places

**Table 8:** Association between reported frequency of use of non-pharmaceutical interventions (NPIs), and frequency of exposure to crowded places, and COVID-19 Illness

	**Unadjusted**		**Model 1**		**Model 2**		**Model 3**	
	**N**	**OR (95% CI)**	**N**	**OR (95% CI)**	**N**	**OR (95% CI)**	**N**	**OR (95% CI)**
Wearing a mask	25,730	**0.74 (0.72 to 0.77)**	22,297	**0.73 (0.70 to 0.76)**	16,843	**0.73 (0.70 to 0.76)**	12,566	**0.66 (0.62 to 0.71)**
Social distancing	25,688	**0.77 (0.74 to 0.80)**	22,257	**0.83 (0.80 to 0.86)**	16,803	**0.88 (0.84 to 0.92)**	12,566	0.96 (0.90 to 1.03)
Handwashing when arriving home	25,844	**0.82 (0.79 to 0.85)**	22,381	**0.84 (0.80 to 0.87)**	16,900	**0.84 (0.80 to 0.88)**	12,566	**0.74 (0.69 to 0.81)**
Handwashing before eating	25,732	**1.13 (1.09 to 1.17)**	22,292	**1.14 (1.10 to 1.18)**	16,849	**1.11 (1.07 to 1.16)**	12,566	**1.13 (1.05 to 1.22)**
Avoid touching face	25,345	**1.10 (1.07 to 1.14)**	21,969	**1.10 (1.06 to 1.14)**	16,636	**1.09 (1.05 to 1.14)**	12,566	1.04 (0.97 to 1.11)
Cleaning things	25,683	**1.26 (1.22 to 1.31)**	22,270	**1.24 (1.19 to 1.29)**	16,819	**1.24 (1.19 to 1.30)**	12,566	**1.34 (1.25 to 1.43)**
Avoid touching others’ pets	21,597	**1.24 (1.21 to 1.28)**	18,726	**1.26 (1.22 to 1.30)**	14,066	**1.27 (1.23 to 1.32)**	12,566	**1.30 (1.24 to 1.37)**
Other	24,272	**1.15 (1.12 to 1.18)**	21,031	**1.14 (1.11 to 1.18)**	15,879	**1.15 (1.11 to 1.19)**	12,566	**1.10 (1.05 to 1.15)**
Been in crowded places 10 people	26,028	**1.16 (1.15 to 1.18)**	22,556	**1.14 (1.12 to 1.15)**	17,027	**1.12 (1.10 to 1.14)**	12,566	**1.09 (1.07 to 1.11)**
Been in crowded places 100 people	25,861	**1.18 (1.15 to 1.20)**	22,401	**1.14 (1.12 to 1.17)**	16,897	**1.12 (1.09 to 1.15)**	12,566	1.04 (1.00 to 1.07)

Model 1: controlling for demographics (age, gender, ethnicity, socioeconomic status), month of questionnaire completion, and vaccination status

Model 2: as per model 1 plus controlling for money problems; working outside home; number of people in the household (lives alone, lives with one other person, 3–6 people in the house, more than 6 people in the house); having pets; pregnancy (assuming missing not pregnant); number of comorbid conditions; history of a mental health problem; self-reported regular use of steroids or immunosuppressant medication; statins; medications for diabetes; self-reported weight (normal, underweight, overweight); smoking status; anxiety (from PHQ-4); depression (from PHQ-4); U.K. region; and month of questionnaire completion interacted with region

Model 3: as per model 2 plus controlling for other NPIs and being in crowded places

Handwashing before eating, avoiding touching the face, cleaning things, avoiding touching pets, and other preventive actions were all associated with an increased odds of COVID-19 infection in all analyses, although some of these were not significant. Being in crowded places of 10–100 people or > 100 people, were both associated with increased odds of COVID-19 infection (10 -100 people 1.62, 95% CI: 1.42 to 1.85 for never vs any and OR 1.12, 95% CI: 1.10 to 1.14 for each increase in level of use; and > 100 people OR 1.73, 95% CI: 1.53 to 1.97 for never vs any and OR 1.12, 95% CI: 1.09 to 1.15 for each increase in level of use, all using model 2).

### Sensitivity analyses

Sensitivity analyses were broadly consistent with the main analyses (Supplementary Table 2).

## Discussion

In this large community-based survey we found strong evidence for a protective effect of wearing a face covering, social distancing and handwashing when arriving home in reducing the risk of COVID-19 infection. We also found strong evidence for an increased risk of COVID-19 infection in those who attended crowded places, with greater risk from attending events with larger (> 100 people) compared with smaller (10–99 people) crowds. We found no evidence of a protective effect from the other non-pharmaceutical interventions under investigation, including those who reported handwashing before eating, cleaning things that might have virus on them, avoiding touching others’ pets, or taking alternative treatments.

Our findings on wearing a face covering, social distancing, and avoiding crowded places, are consistent with current evidence about airborne transmission of SARS-CoV-2, [12] and are already widely accepted as useful public health measures. Our finding of the strongest effects for wearing a face covering significantly add to the limited prior evidence on the effects of face masks in community settings [4, 8] and support the role of face mask use in reducing the risk for the wearer of the mask and not just for contacts. However, most experts believe that fomite transmission plays a minimal role in transmission [13, 14], and therefore our finding of an association between handwashing on arriving home and reduced COVID-19 infection is important. The increased odds of COVID-19 infection associated with many of the NPIs under investigation in this study was an unexpected and surprising finding. We have not been able to identify a plausible biological mechanism through which these NPIs could increase the risk of infection. These findings most likely result from bias or uncontrolled confounding. It is possible that some of these behaviours (avoiding touching others pets for example) are associated with leaving the house and therefore being exposed to more potentially infected people. The most likely cause is probably recall bias – participants who had an infection were more likely to perceive increased use of a NPI than those who did not. More than a third of our participants reported avoiding touching their face always or very often, but other studies have reported that face-touching is very common [15]. However, if recall bias is relevant then it is likely to affect all the NPIs that we asked about, so the reduced odds of infection associated with wearing a face covering, social distancing, handwashing when arriving home and avoiding crowds are likely to be under-estimates of the true effects.

Systematic reviews and meta-analyses of NPIs to reduce the incidence of COVID-19 are consistent with our findings [4, 8, 9]. A review of 172 observational studies published in the Lancet in 2020 reported pooled adjusted odds ratios of 0.15, 95% CI: 0.07 to 0.34 for use of face masks and 0.18, 95% CI 0.09 to 0.38 for social distancing (our model 2 ORs were 0.19 and 0.35 for mask wearing and social distancing respectively [4]. A subsequent systematic review and meta-analysis of 72 studies reported relative risks of 0.47, 95% CI: 0.29 to 0.75 and 0.75, 95% CI: 0.59 to 0.95 for face mask use and social distancing respectively [9]. These are slightly smaller than the effect sizes in our study and the previous review, but demonstrate a consistent pattern of an important effect with mask wearing being slightly stronger than social distancing. The results of the current study add significantly to the prior literature given most studies included in the prior reviews were small, the limited control of confounding, and the settings (commonly secondary care or less typical community samples such as from high risk gatherings or travellers) [4, 8, 9]. A Cochrane review of physical interventions to reduce the spread of respiratory viruses, that was not restricted to COVID-19 and included only trial data, reported that wearing a face mask may make little or no difference to incidence of influenza-like illness [16]. However, they acknowledged that there was low compliance to the intervention and they were not able to draw firm conclusions or generalise the findings to the COVID-19 pandemic. A recent trial evaluated advice to use a face mask in 4862 participants during April and May 2020 [17]. The difference in SARS-CoV-2 infection was not statistically significant but the confidence interval was compatible with anywhere from a 46% reduction to 32% increase. The more recent review also found a non-significant 53% reduction (relative risk 0.47, 0.19 to 1.12) for handwashing [9]. This is comparable to our primary analysis estimates for handwashing on arriving home (model 2 OR 0.63, 95% CI: 0.48 to 0.83). It is worth noting that our effect estimates for all these NPIs showed evidence of a dose–response effect, with increases in effect for each increase in the frequency of use.

An ecological study looking at transmission rates and public health measures in 190 countries found that the largest reduction in time-varying effective reproduction number (Rt) was associated with social distancing (− 42.94%, − 44.24% to − 41.60%), while smaller, but still important, reductions were associated with mandatory use of face masks (− 15.14%, − 21.79% to − 7.93%) and quarantine policies (− 11.40%, − 13.66% to − 9.07%) [18].

Our finding that handwashing when arriving home appears to be associated with reduced risk, but handwashing before meals is associated with increased (or more likely no reduction) in risk, is interesting and suggests that fomite transmission is important when people travel outside of their home. Transmission of COVID-19 does frequently occur within households. Our analysis was not designed to look specifically at household transmission and so excluded people with a household contact, which is where any preventive effect of handwashing before meals is likely to occur [19]. However, our data would suggest that washing hands when arriving home is more likely to be effective at reducing the risk of COVID-19 coming into the household. We are not aware of any other studies that have compared the effects of handwashing at these different times.

A recent narrative review focusing on factors that influence engagement with NPIs found that women, more highly educated people, older people, married people, and those with worse self-rated health were more likely to engage with use of face masks [20]. Our analyses adjusted for most of these factors, although we did not adjust for marital status or ‘self-rated health’. More generally, there was widespread acceptance of the need to adopt NPIs. Perceived severity of the pandemic and personal risk were key factors influencing willingness to adhere. Interestingly, a narrative review conducted several years before the pandemic found greater perceived willingness to accept measures like handwashing and ‘respiratory hygiene’ than mask wearing and personal distancing [21].

Strengths of our study include our broad and inclusive approach to recruitment, large number of participants, rigorous criteria for defining COVID-19 illness, and detailed data on sociodemographic and medical factors that could be controlled for in the model. Community testing for COVID-19 was virtually non-existent in the UK at the start of the pandemic, and only became widely available in the autumn of 2020. Therefore, we decided to broaden the case definition used for our primary analysis beyond having a positive test for COVID-19. However, we took a rigorous approach, including only those without a positive test result if they had a respiratory illness during the pandemic that was associated with both fever AND loss of smell or taste, and this ended up being the minority of cases in our primary analysis. We also conducted sensitivity analyses using a broader symptomatic definition and with any ARI lasting three days or more. A substantial proportion of those who reported having a positive COVID-19 test did not report having an ARI lasting three days or more, but we did not ask about ARI symptoms lasting less than three days so it is not possible to tell how many of these were asymptomatic.

The main weakness of our study is that we used retrospective self-reported data. Almost all studies of public health measures are observational and use self-report data as it is very difficult to randomise people to follow (and continue to adhere to) different public health measures. We have already discussed the risk of recall bias associated with this approach, but we believe that recall bias is unlikely to explain the reduced risk of COVID-19 in those who described handwashing on arriving home, wearing a face mask and social distancing, or the difference in risk between handwashing on arriving home and handwashing before meals. Confounding is the other major risk associated with observational studies such as this. However, we were able to measure and adjust for all key known confounders and many other potential confounders. Although we cannot exclude some residual confounding, this is unlikely to explain the large effects observed in our study. Our study sample included a larger proportion of females (62.0% vs 51.1%) and adults aged 50–64 (32.8% vs 24.5%) and 65–79 (27.4% vs 17.2%) than the general UK population. We also had an over-representation of people from white ethnic groups and under-representation from other ethnic groups. Nevertheless, we were able to control for these characteristics in our analyses. The “other behaviours” in this analysis includes a heterogeneous group of behaviours, including exercise, nutritional supplements and herbal remedies. It is possible that some of these behaviours may be associated with reduced odds of COVID-19 when explored individually.

## Conclusions

These data add to the growing body of evidence for the importance of wearing a face covering and social distancing (including avoiding crowded places) in reducing the risk of transmission of COVID-19. We also found evidence supporting the use of handwashing upon returning home. We specifically excluded participants where there had been COVID-19 infection in the home, so this data reflects infections coming into the household and not transmission within households. Nevertheless, for incoming infections we found no evidence supporting use of handwashing before eating, avoiding touching the face, cleaning things with virus on, or avoiding touching other people’s pets. Given the strength of the associations found in this study, the historical evidence for the beneficial effects of handwashing, and the low risk of serious harm from promoting such an approach, it would seem prudent to encourage increased uptake of handwashing on arriving home based on these findings.

## Supplementary Information

**Additional file 1: Supplementary Table 1.** Characteristics primary analysis population compared with excluded and ‘COVID-19 without ARI’ populations. **Supplementary Table 2.** Sensitivity analyses.

## Data Availability

The datasets used and/or analysed during the current study are available from the corresponding author on reasonable request.

## Abbreviations

CI: Confidence Interval
NPI: Non-pharmaceutical interventions
OR: Odds ratio
PHQ-4: Patient Health Questionnaire-4
RTO-COVID-19: Retrospective Treatment and Outcomes study on COVID-19
SARS-CoV-2: Severe acute respiratory syndrome coronavirus 2
U.K.: United Kingdom

## References

[1] del Rio C, Omer SB, Malani PN (2022). Winter of omicron—the evolving COVID-19 pandemic. JAMA.

[2] Buitrago-Garcia D, Egli-Gany D, Counotte MJ, Hossmann S, Imeri H, Ipekci AM (2020). Occurrence and transmission potential of asymptomatic and presymptomatic SARS-CoV-2 infections: a living systematic review and meta-analysis. PLOS Med.

[3] Meyerowitz EA, Richterman A, Gandhi RT, Sax PE (2021). Transmission of SARS-CoV-2: A Review of Viral, Host, and Environmental Factors. Ann Intern Med.

[4] Chu DK, Akl EA, Duda S, Solo K, Yaacoub S, Schünemann HJ (2020). Physical distancing, face masks, and eye protection to prevent person-to-person transmission of SARS-CoV-2 and COVID-19: a systematic review and meta-analysis. The Lancet.

[5] Wang Y, Tian H, Zhang L, Zhang M, Guo D, Wu W (2020). Reduction of secondary transmission of SARS-CoV-2 in households by face mask use, disinfection and social distancing: a cohort study in Beijing, China. BMJ Glob Health.

[6] Shi J, Wen Z, Zhong G, Yang H, Wang C, Huang B (2020). Susceptibility of ferrets, cats, dogs, and other domesticated animals to SARS–coronavirus 2. Science.

[7] Timeline of UK government coronavirus lockdowns and restrictions. The Institute for Government. 2021 [cited 2022 Apr 14]. Available from: https://www.instituteforgovernment.org.uk/charts/uk-government-coronavirus-lockdowns

[8] Chou R, Dana T, Jungbauer R, Weeks C, McDonagh MS (2020). Masks for prevention of respiratory virus infections, including SARS-CoV-2, in health care and community settings. Ann Intern Med.

[9] Talic S, Shah S, Wild H, Gasevic D, Maharaj A, Ademi Z (2021). Effectiveness of public health measures in reducing the incidence of covid-19, SARS-CoV-2 transmission, and covid-19 mortality: systematic review and meta-analysis. BMJ.

[10] Dixon BE, Wools-Kaloustian KK, Fadel WF, Duszynski TJ, Yiannoutsos C, Halverson PK (2021). Symptoms and symptom clusters associated with SARS-CoV-2 infection in community-based populations: results from a statewide epidemiological study. PLoS ONE.

[11] World Health Organisation. WHO COVID-19 Case definition. [cited 2021 May 24]. Available from: https://www.who.int/publications-detail-redirect/WHO-2019-nCoV-Surveillance_Case_Definition-2020.2

[12] Cevik M, Kuppalli K, Kindrachuk J, Peiris M (2020). Virology, transmission, and pathogenesis of SARS-CoV-2. BMJ.

[13] Mondelli MU, Colaneri M, Seminari EM, Baldanti F, Bruno R (2021). Low risk of SARS-CoV-2 transmission by fomites in real-life conditions. Lancet Infect Dis.

[14] Kampf G, Brüggemann Y, Kaba HE, Steinmann J, Pfaender S, Scheithauer S (2020). Potential sources, modes of transmission and effectiveness of prevention measures against SARS-CoV-2. J Hosp Infect..

[15] Rahman J, Mumin J, Fakhruddin B. How frequently do we touch facial T-Zone: a systematic review. Ann Glob Health. 2020;86(1). 10.5334/aogh.2956 PMC7350942 32704480

[16] Jefferson T, Mar CBD, Dooley L, Ferroni E, Al-Ansary LA, Bawazeer GA, et al. Physical interventions to interrupt or reduce the spread of respiratory viruses. Cochrane Database Syst Rev. 2020 [cited 2022 Dec 19];(11). Available from: https://www.cochranelibrary.com/cdsr/doi/10.1002/14651858.CD006207.pub5/full 10.1002/14651858.CD006207.pub5 PMC8094623 33215698

[17] Bundgaard H, Bundgaard JS, Raaschou-Pedersen DET, von Buchwald C, Todsen T, Norsk JB (2021). Effectiveness of adding a mask recommendation to other public health measures to prevent SARS-CoV-2 infection in danish mask wearers. Ann Intern Med.

[18] Bo Y, Guo C, Lin C, Zeng Y, Li HB, Zhang Y (2021). Effectiveness of non-pharmaceutical interventions on COVID-19 transmission in 190 countries from 23 January to 13 April 2020. Int J Infect Dis.

[19] Little P, Stuart B, Hobbs FDR, Moore M, Barnett J, Popoola D (2015). An internet-delivered handwashing intervention to modify influenza-like illness and respiratory infection transmission (PRIMIT): a primary care randomised trial. The Lancet.

[20] Seale H, Dyer CEF, Abdi I, Rahman KM, Sun Y, Qureshi MO (2020). Improving the impact of non-pharmaceutical interventions during COVID-19: examining the factors that influence engagement and the impact on individuals. BMC Infect Dis.

[21] Teasdale E, Santer M, Geraghty AWA, Little P, Yardley L (2014). Public perceptions of non-pharmaceutical interventions for reducing transmission of respiratory infection: systematic review and synthesis of qualitative studies. BMC Public Health.

