# Supplementary material for: Non-pharmaceutical interventions and risk of COVID-19 infection: survey of U.K. public from November 2020 – May 2021
Source: BMC Public Health. 2023 Feb 24;23:389. doi: 10.1186/s12889-023-15209-6 (PMC9951136; doi:10.1186/s12889-023-15209-6)
Supplement: Supplementary file 1 — Additional file 1: Supplementary Table 1. Characteristics primary analysis population compared with excluded and ‘COVID-19 without ARI’ populations. Supplementary Table 2. Sensitivity analyses. [file 12889_2023_15209_MOESM1_ESM.docx]

**Supplementary Materials**

**Supplementary table 1: Characteristics primary analysis population compared with excluded and ‘COVID-19 without ARI’ populations**

|  | Primary analysis population  N=27758 | | Excluded - household contact  N=3746 | | Excluded – possible but not probable COVID-19  N= 2130 | | COVID-19 positive but not reporting ARI population  N=1175 | |
| --- | --- | --- | --- | --- | --- | --- | --- | --- |
|  | n | % | n | % | n | % | n | % |
| Gender |  |  |  |  |  |  |  |  |
| Female | 17064 | 62.0 | 2507 | 67.7 | 1470 | 70.0 | 792 | 67.9 |
| Male | 10467 | 38.0 | 1199 | 32.4 | 630 | 30.0 | 374 | 32.1 |
| Age group |  |  |  |  |  |  |  |  |
| <25 | 1552 | 5.6 | 397 | 10.7 | 151 | 7.1 | 105 | 9.0 |
| 25-49 | 8754 | 31.8 | 1799 | 48.3 | 890 | 42.0 | 542 | 46.5 |
| 50-64 | 9044 | 32.8 | 1102 | 29.6 | 700 | 33.0 | 386 | 33.1 |
| 65-79 | 7555 | 27.4 | 408 | 11.0 | 362 | 17.1 | 128 | 11.0 |
| 80+ | 639 | 2.3 | 17 | 0.5 | 19 | 0.9 | 5 | 0.4 |
| Ethnicity |  |  |  |  |  |  |  |  |
| White | 23169 | 92.3 | 3146 | 88.2 | 1686 | 92.3 | 834 | 86.5 |
| Black | 389 | 1.6 | 65 | 1.8 | 25 | 1.4 | 33 | 3.4 |
| South Asian | 752 | 3.0 | 200 | 5.6 | 53 | 2.9 | 71 | 7.4 |
| Chinese | 119 | 0.5 | 13 | 0.4 | 5 | 0.3 | 2 | 0.2 |
| Arab | 70 | 0.3 | 13 | 0.4 | 5 | 0.3 | 3 | 0.3 |
| Mixed | 356 | 1.4 | 79 | 2.2 | 26 | 1.4 | 14 | 1.5 |
| Don't know | 22 | 0.1 | 3 | 0.1 | 2 | 0.1 | 3 | 0.3 |
| No answer | 67 | 0.3 | 10 | 0.3 | 4 | 0.2 | 1 | 0.1 |
| Other | 151 | 0.6 | 39 | 1.1 | 20 | 1.1 | 3 | 0.3 |
| Money problems |  |  |  |  |  |  |  |  |
| No problems | 18154 | 72.6 | 2183 | 61.4 | 1185 | 65.2 | 597 | 62.1 |
| Some problems | 5676 | 22.7 | 1105 | 31.1 | 495 | 27.2 | 311 | 32.3 |
| Big problems | 808 | 3.2 | 172 | 4.8 | 82 | 4.5 | 37 | 3.9 |
| Huge problems | 362 | 1.5 | 95 | 2.7 | 55 | 3.0 | 17 | 1.8 |
| Work outside home |  |  |  |  |  |  |  |  |
| No | 20496 | 81.5 | 2769 | 77.5 | 1486 | 81.4 | 584 | 60.3 |
| Yes | 4643 | 18.5 | 802 | 22.5 | 340 | 18.6 | 384 | 39.7 |
| Number in household |  |  |  |  |  |  |  |  |
| Live alone | 8513 | 42.7 | 603 | 19.2 | 617 | 38.9 | 242 | 29.2 |
| Live with one other | 4612 | 23.1 | 642 | 20.2 | 327 | 20.6 | 165 | 19.9 |
| 3-6 people | 6146 | 30.8 | 1605 | 52.5 | 575 | 36.3 | 356 | 42.9 |
| 7 or more | 690 | 3.5 | 250 | 8.0 | 66 | 4.2 | 67 | 8.1 |

**Supplementary Table 2: Sensitivity analyses**

| **Sensitivity analysis** | **1. Exclude vaccinated** | | **2. Include possible Covid** | | **3. Symptomatic covid controlling for exposure window** | **4. ARI controlling for exposure window** | |
| --- | --- | --- | --- | --- | --- | --- | --- |
|  | **Unadjusted** | **Adjusted** | **Unadjusted** | **Adjusted** | **Adjusted** | **Unadjusted** | **Adjusted** |
| NPI |  | N=3069 |  | N=13404 | N=21608 |  | N=10930 |
| Wearing a mask | **0.81 (0.76 to 0.86)** | **0.79(0.74 to 0.85)** | **0.60 (0.59 to 0.62)** | **0.59 (0.57 to 0.61)** | **0.47 (0.45 to 0.50)** | **0.54 (0.52 to 0.55)** | **0.50 (0.48 to 0.52)** |
| Social distancing | **0.87 (0.81 to 0.93)** | **0.88 (0.82 to 0.94)** | **0.69 (0.67 to 0.71)** | **0.73 (0.71 to 0.75)** | **0.63 (0.60 to 0.66)** | **0.68 (0.66 to 0.69)** | **0.72 (0.69 to 0.74)** |
| Handwashing when arriving home | **0.89 (0.84 to 0.96)** | **0.90 (0.83 to 0.96)** | **0.72 (0.70 to 0.74)** | **0.73 (0.70 to 0.75)** | **0.64 (0.61 to 0.67)** | **0.70 (0.68to 0.71)** | **0.68 (0.66 to 0.70)** |
| Handwashing before eating | **1.19 (1.11 to 1.26)** | **1.18 (1.11 to 1.27)** | **1.04 (1.02 to 1.07)** | **1.06 (1.03 to 1.09)** | 1.02 (0.98 to 1.06) | **1.01 (0.99 to 1.04)** | 1.02 (0.99 to 1.05) |
| Avoid touching face | **1.10 (1.03 to 1.17)** | **1.09 (1.02 to 1.16)** | 0.98 (0.95 to 1.00) | 0.97 (0.95 to 1.00) | **0.89 (0.85 to 0.93)** | **0.96 (0.93 to 0.98)** | **0.95 (0.92 to 0.98)** |
| Cleaning things | **1.23 (1.16 to 1.31)** | **1.22 (1.14 to 1.30)** | **1.10 (1.07 to 1.12)** | **1.08 (1.05 to 1.12)** | 1.01 (0.97 to 1.06) | **1.06 (1.04 to 1.09)** | **1.06 (1.03 to 1.10)** |
| Avoid touching others pets | **1.25 (1.18 to 1.32)** | **1.25 (1.17 to 1.33)** | **1.14 (1.11 to 1.16)** | **1.15 (1.12 to 1.18)** | 1.02 (0.98 to 1.07) | **1.13 (1.11 to 1.15)** | **1.13 (1.10 to 1.16)** |
| Other | **1.17 (1.11 to 1.24)** | **1.16 (1.10 to 1.23)** | **1.08 (1.06 to 1.11)** | **1.07 (1.05 to 1.10)** | 0.98 (0.95 to 1.03) | **1.08 (1.06 to 1.10)** | **1.07 (1.04 to 1.10)** |
| Been in crowded places 10 people | **1.15 (1.12 to 1.18)** | **1.15 (1.12 to 1.18)** | **1.19 (1.17 to 1.20)** | **1.17 (1.16 to 1.18)** | **1.23 (1.21 to 1.25)** | **1.19 (1.18 to 1.20)** | **1.17 (1.16 to 1.19)** |
| Been in crowded places 100 people | **1.15 (1.11 to 1.19)** | **1.16 (1.11 to 1.20)** | **1.22 (1.20 to 1.23)** | **1.19 (1.17 to 1.21)** | **1.26 (1.23 to 1.30)** | **1.26 (1.24 to 1.28)** | **1.23 (1.20 to 1.25)** |
